# Asymmetric azide-alkyne Huisgen cycloaddition on chiral metal surfaces

**DOI:** 10.1038/s42004-021-00488-0

**Published:** 2021-04-12

**Authors:** Samuel Stolz, Michael Bauer, Carlo A. Pignedoli, Nils Krane, Max Bommert, Elia Turco, Nicolò Bassi, Amogh Kinikar, Néstor Merino-Dìez, Roland Hany, Harald Brune, Oliver Gröning, Roland Widmer

**Affiliations:** 1 Empa, Swiss Federal Laboratories for Materials Science and Technology, Nanotech@Surfaces Laboratory, Dübendorf, Switzerland; 2 Institute of Physics, École Polytechnique Fédérale de Lausanne, Lausanne, Switzerland; 3 Empa, Swiss Federal Laboratories for Materials Science and Technology, Laboratory for Functional Polymers, Dübendorf, Switzerland

**Keywords:** Asymmetric catalysis, Heterogeneous catalysis, Scanning probe microscopy

## Abstract

Achieving fundamental understanding of enantioselective heterogeneous synthesis is marred by the permanent presence of multitudinous arrangements of catalytically active sites in real catalysts. In this study, we address this issue by using structurally comparatively simple, well-defined, and chiral intermetallic PdGa{111} surfaces as catalytic substrates. We demonstrate the impact of chirality transfer and ensemble effect for the thermally activated azide-alkyne Huisgen cycloaddition between 3-(4-azidophenyl)propionic acid and 9-ethynylphenanthrene on these threefold symmetric intermetallic surfaces under ultrahigh vacuum conditions. Specifically, we encounter a dominating ensemble effect for this reaction as on the Pd_3_-terminated PdGa{111} surfaces no stable heterocoupled structures are created, while on the Pd_1_-terminated PdGa{111} surfaces, the cycloaddition proceeds regioselectively. Moreover, we observe chirality transfer from the substrate to the reaction products, as they are formed enantioselectively on the Pd_1_-terminated PdGa{111} surfaces. Our results evidence a determinant ensemble effect and the immense potential of PdGa as asymmetric heterogeneous catalyst.

## Abstract (web summary)

Mechanistic insight into enantioselective reactions at intrinsically chiral surfaces can be challenging to obtain. Here the catalytic activity of Pd_1_- and Pd_3_-terminated PdGa{111} surfaces is shown to differ substantially, with Pd_1_-terminated surfaces promoting on-surface azide– alkyne cycloadditions enantioand regioselectively.

## Introduction

In recent years, on-surface synthesis under ultrahigh vacuum (UHV) conditions has emerged as a very successful method to produce extended, covalently bonded macro-molecules, which are inaccessible to wet-chemical synthesis due to insolubility or high reactivity^1^. On-surface synthesis is based on the controlled reaction of dedicated precursor molecules on well-defined single-crystal surfaces mostly by thermal activation. Although in recent years, the catalog of on-surface reactions and synthesized structures broadened extensively, the focus rarely lay on enantioselective synthesis, which is of immense importance for instance in pharmaceutical, agricultural, or food industry^2,3^. This lack of enantioselective on-surface synthesis originates largely from the scarcity of intrinsically chiral, catalytically active, and well-characterized single-crystal surfaces needed for this task. One option to circumvent this deficiency is to render achiral surfaces chiral via the adsorption of enantiopure molecules^4–10^. This approach has proven very successful under ambient conditions, for instance, in the asymmetric hydrogenation of activated ketones and asymmetric hydrogenation of prochiral olefins^11^.

Intrinsically chiral metal surfaces promise an increased thermal stability, a reduction of complexity arising from the multitude of molecule–molecule arrangements and interactions, and would not require enantiopure molecular modifiers, but are accompanied by a reduced adjustability towards specific reactions. In this context, high Miller-index surfaces of achiral crystals^12,13^, which exhibit a low density of chiral centers only at the kink sites of atomic steps separating adjacent terraces of low-index surfaces, have been established for enantioselective decomposition reactions under UHV conditions^14,15^.

**Fig. 1:**
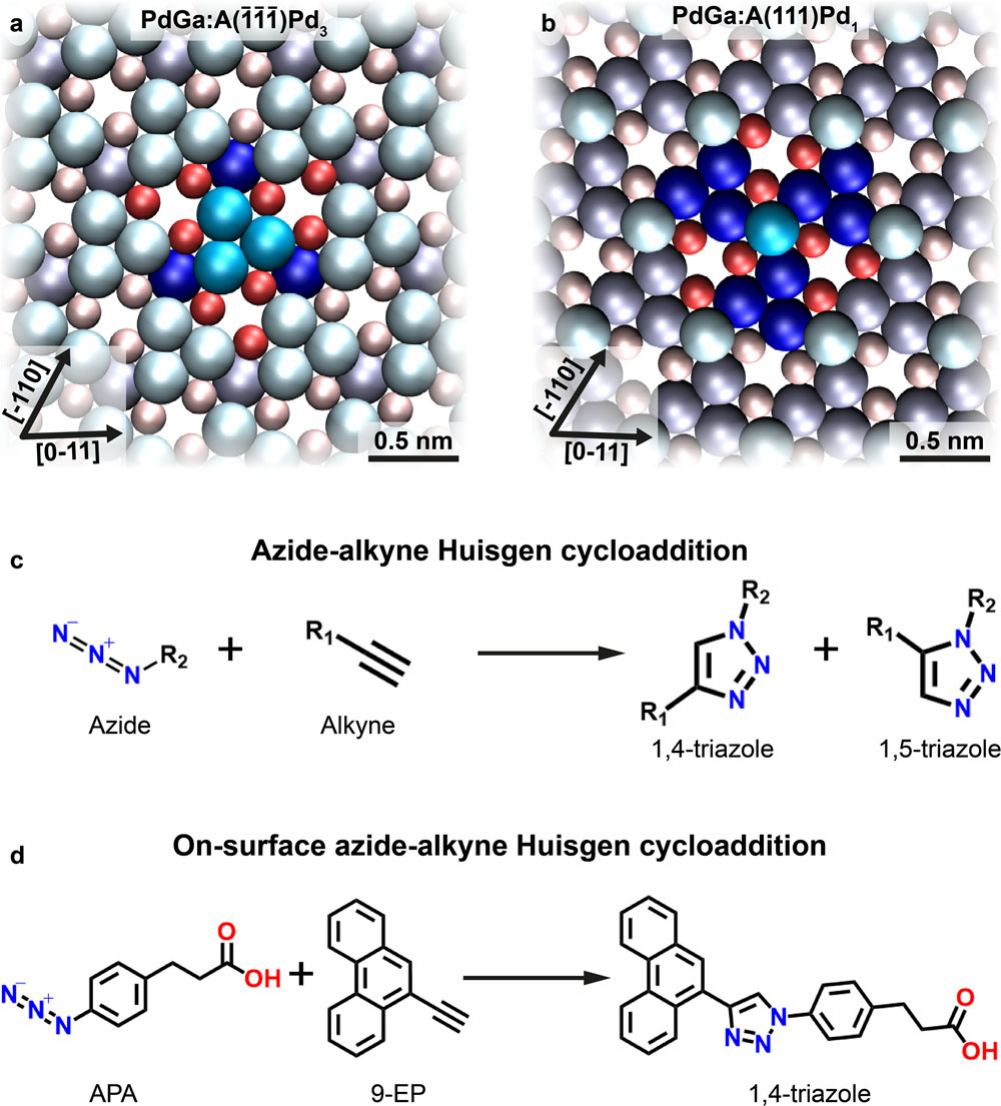
Schematics of the PdGa{111} surfaces and the azide–alkyne Huisgen cycloaddition. The atomic surface structure of **a** PdGa:A($\bar 1\bar 1\bar 1$)Pd_3_ (first layer Pd_3_ in bright blue: *z* = 0 pm; second layer Ga_3_ in red: *z* = −85 pm; third layer Pd_1_ in dark blue: *z* = −161 pm) and **b** PdGa:A(111)Pd_1_ (first layer Pd_1_ in bright blue: *z* = 0 pm; second layer Ga_3_ in red: −57.4 pm; third layer Pd_3_ in dark blue: −149.8 pm) with their chirality highlighted by a top layer Pd trimer or a single top layer atom, respectively, and their neighbors in saturated colors. **c** General reaction scheme of the azide–alkyne Huisgen cycloaddition. **d** The investigated on-surface Huisgen cycloaddition between 9-ethynylphenanthrene (9-EP) and 3-(4-azidophenyl)propionic acid (APA) yielding 1,4-triazole regioisomers.

Low Miller-index surfaces of intrinsically chiral bulk crystals possess a high density of well-defined and thermally stable chiral centers. Even though chiral metallic crystals gain increasing attention in the context of topological electronic properties^16–23^, in-depth characterization of their surface structure is very limited. Currently, the only intrinsically chiral and catalytically active single crystal whose low-index surfaces are well-characterized is intermetallic PdGa. PdGa belongs to the non-centrosymmetric space-group P2_1_3 (ref. ^24^), and thus exists in two enantiomorphs, denoted as PdGa:A and PdGa:B^25^. Here, we focus on the two structurally dissimilar, bulk-truncated, threefold symmetric PdGa:A($\bar 1\bar 1\bar 1$) and PdGa:A(111) surfaces, of which the former is terminated by isolated Pd trimers, further referred to as A:Pd_3_ (Fig. 1a), while the top layer of the latter consists of single, isolated Pd atoms, and is denoted A:Pd_1_ (Fig. 1b)^26^. Owing to their differing surface terminations, in combination with their equal lattice parameters, identical symmetry, and similar electronic properties, the PdGa{111} surfaces are ideally suited to disentangle ensemble and ligand effects, i.e., the influence of the local geometric and electronic properties, respectively, in asymmetric heterogeneous catalysis^27–30^.

Chirality transfer from the PdGa{111} surfaces onto molecular processes has been demonstrated with acetylene (C_2_H_2_; achiral) and 9-ethynylphenanthrene (9-EP; prochiral, thus appearing in two distinguishable surface enantiomers R and S when confined to a planar configuration)^31,32^. Specifically, C_2_H_2_ has been reported to exhibit directed rotation on the Pd_3_-terminated PdGa{111} surfaces^31^. On the other hand, for 9-EP, enantiopure trimerization from an initial racemic mixture of monomers on the Pd_3_- and enantioselective adsorption of individual monomers on the Pd_1_-terminated PdGa{111} surfaces have been shown^32^.

Therefore, 9-EP promotes itself as prototypical alkyne precursor for investigating enantioselective synthesis on the PdGa{111} surfaces. Moreover, on-surface azide–alkyne Huisgen cycloaddition (Fig. 1c) without emphasis on enantioselectivity has been successfully demonstrated on Au(111)^33^, and, using 9-EP as precursor, on Cu(111) under UHV conditions by Bebensee et al.^34^. This catalytically activated reaction, which belongs to the class of Click Chemistry, selectively yields 1,4-triazole regioisomers and omits the formation of 1,5-triazoles. According to the d-band model introduced by Liu and Nørskov^28^ and Nørskov et al.^35^, PdGa is expected to possess similar catalytic activity for the azide–alkyne Huisgen cycloaddition as copper. To perform the reaction, we chose commercially available prochiral 3-(4-azidophenyl)propionic acid (APA) as azide reactant (Fig. 1d).

Using scanning tunneling microscopy (STM), complemented with X-ray photoelectron spectroscopy (XPS), and density functional theory (DFT) calculations, we demonstrate a significant ensemble effect on the PdGa{111} surface reactivity, manifested in the occurrence of the regio- and, most importantly, enantioselective cycloaddition between APA and 9-EP on the Pd_1_-terminated PdGa{111} surfaces, but its suppression on Pd_3_-terminated PdGa{111}.

## Results and discussion

### Azide–alkyne Huisgen cycloaddition on Pd_3_-terminated PdGa{111} surfaces

**Fig. 2:**
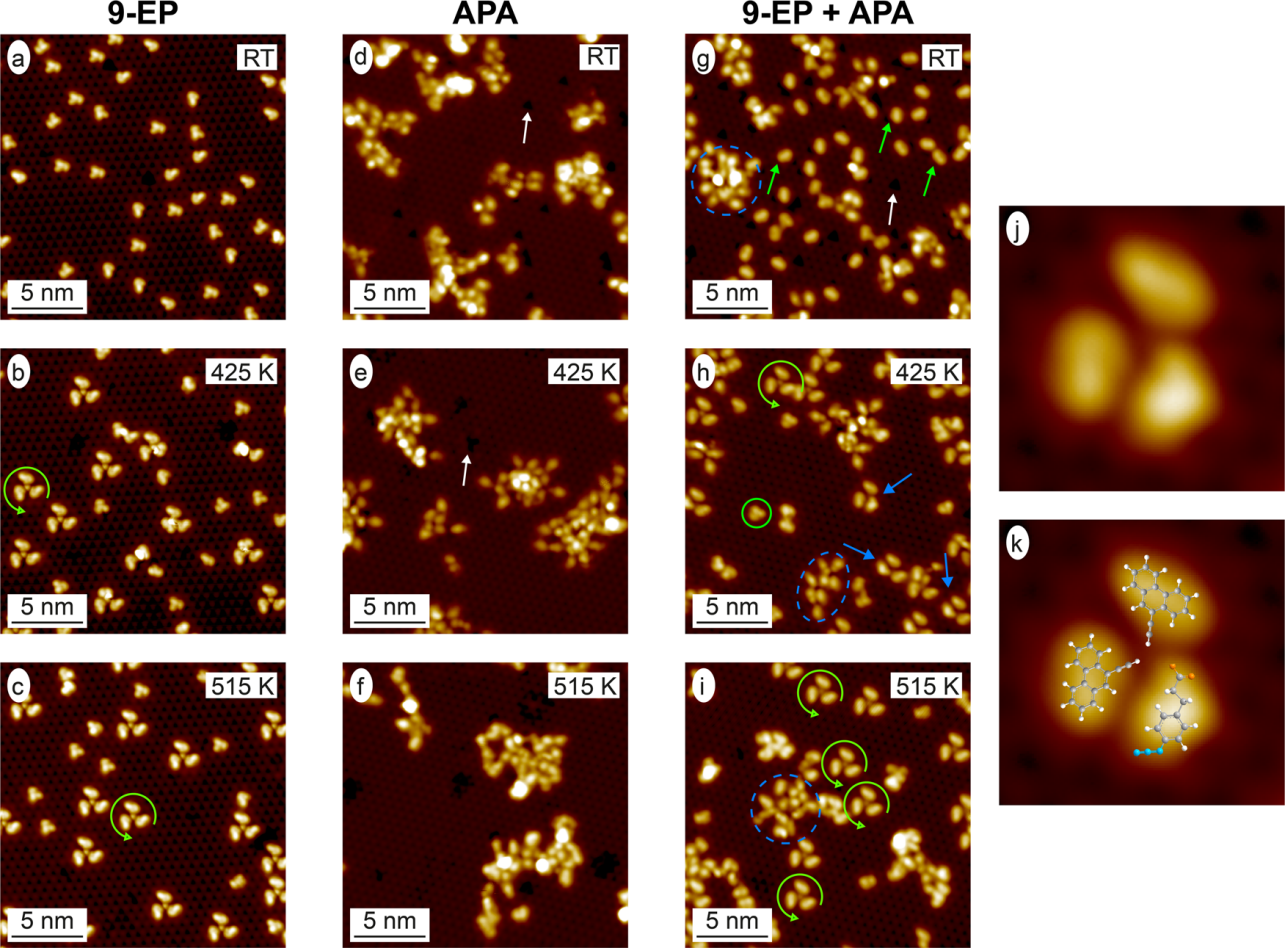
Suppressed azide–alkyne Huisgen cycloaddition on the Pd_3_-terminated PdGa{111} surfaces. STM images (*V*_B_ = 20 mV; *I*_T_ = 1 nA) of 9-EP **a** deposited on PdGa:A($\bar 1\bar 1\bar 1$)Pd_3_ at RT, and after subsequent annealing to **b** 425 K and **c** 515 K. STM images (*V*_B_ = 200 mV; *I*_T_ = 0.1 nA) of APA molecules on the PdGa:A($\bar 1\bar 1\bar 1$)Pd_3_ surface after **d** RT deposition, subsequent annealing to **e** 425 K and **f** 515 K. STM images (*V*_B_ = 200 mV; *I*_T_ = 0.1 nA) of APA co-adsorbed with 9-EP after **g** RT deposition, subsequent annealing to **h** 425 K and **i** 515 K. High-resolution STM image (3 × 3 nm^2^; *V*_B_ = 20 mV; *I*_T_ = 0.2 nA) of the frequently observed molecular structure containing one APA and two 9-EP molecules, which is marked with blue arrows in **h**, is shown in **j** and overlaid with the molecular structure in **k**. The green circular arrows in **b**, **c**, **h**, **i** highlight the homochiral 9-EP propeller, the white arrows in **d**, **e**, **g** the depression caused by APA remnants, and the green arrows in **g** a 9-EP molecule interacting with an APA remnant. The blue dashed circles in **g**, **h**, **i** indicate APA agglomerations and the green circle in **h** a 9-EP monomer.

A series of STM images of the individual deposition of 9-EP and APA molecules and the co-deposition of both molecules on A:Pd_3_ at room temperature (RT) and after subsequent annealing to 425 and 515 K is shown in Fig. 2. As previously reported^32^, 9-EP deposited at RT results in a homogeneous racemate of well isolated monomers (Fig. 2a) and formation of 9-EP trimers with increasing enantiomeric excess (ee) up to 97 ± 2% towards RRR enantiomorphs for increasing temperatures (Fig. 2b, c). On the other hand, APA molecules deposited on A:Pd_3_ at RT create large, disordered agglomerates (Fig. 2d). Additionally, depressions, which are absent on the pristine surface, are observed on the substrate, as indicated by the white arrow. We attribute these depressions to fragments of the decomposed carboxylic acid group of the APA molecule as they are also observed upon deposition of ex situ synthesized 1,4-triazoles (Fig. S1) and XPS investigations show a strong C 1*s* component arising from the carboxylic acid group (Fig. S2a, Table S1 and Supplementary Note 1). On the other hand, the ratio between the C 1*s* and N 1*s* XPS intensity is around three times larger than the one expected for pristine APA (Fig. S3 and Table S4). Hence also most azide groups decompose, and desorb from the surface. Subsequent annealing of the sample up to 515 K leads to a reduction of the depressions, as they desorb, while agglomeration proceeds without ever forming discernible regular structures, i.e., reoccurring structures of similar topographic signature in STM images (Fig. 2d–f).

When APA is co-deposited with 9-EP on A:Pd_3_, no intermolecular reactions are observed at RT. While APA again agglomerates, 9-EP seems to mostly bond to the previously mentioned molecular fragments detached from APA molecules (depressions), as pointed out by the green arrow in Fig. 2g. Upon annealing to 425 K (Fig. 2h), 9-EP appears in its pristine adsorption configuration, which means it is detached from the molecular fragments, and, importantly, new prochiral 9-EP/APA heterostructures that consist of two 9-EP and one APA molecule are formed (Fig. 2j–k). The adsorption configuration of 9-EP molecules incorporated in these trimer heterostructures with respect to the PdGa substrate is identical with that of R enantiomers incorporated into pure 9-EP trimers^32^. The heterostructure trimers exclusively appear in the enantiomeric form shown in Fig. 2j, k on the A:Pd_3_ termination. The incorporated APA is not covalently bound in these heterostructure trimers, as, upon further annealing to 515 K, these structures deplete and homostructural 9-EP trimers become the only regular motive (highlighted by a green circular arrow in Fig. 2i). Therefore, even though PdGa is copper-like with regard to the catalytic reactivity according to the d-band model^35^, the azide–alkyne Huisgen cycloaddition cannot be triggered with any significant yield on the A:Pd_3_ surface.

### Azide–alkyne Huisgen cycloaddition on Pd_1_-terminated PdGa{111} surfaces

**Fig. 3:**
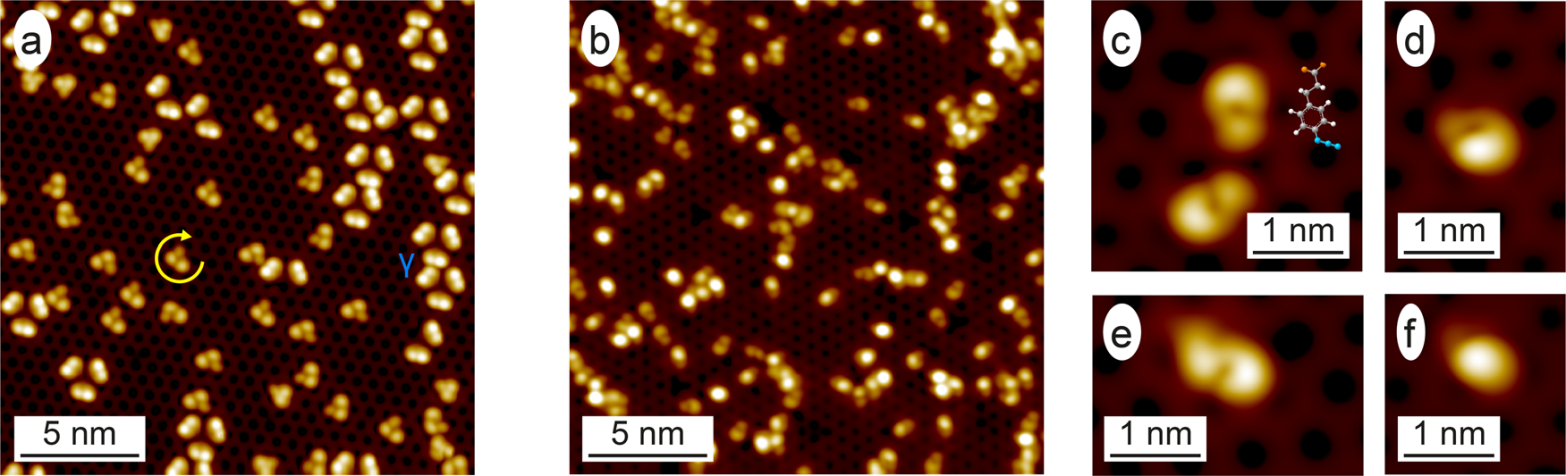
Alkyne and azide on the Pd_1_-terminated PdGa{111} surfaces. Large-scale STM images of **a** 9-EP (*V*_B_ = 20 mV; *I*_T_ = 2 nA) and **b** APA (*V*_B_ = 100 mV; *I*_T_ = 0.4 nA) deposited at RT on PdGa:A(111)Pd_1_. **c**–**f** High-resolution STM images (*V*_B_ = 20 mV; *I*_T_ = 0.2 nA) of the different APA configurations on PdGa:A(111)Pd_1_ with the molecular structure of APA in **c**.

On the A:Pd_1_ surface, 9-EP deposited at RT adsorbs with an ee of 98% in favor of the R enantiomer (cf. Fig. 3a), and dimerizes without ee above 400 K, as reported by Prinz et al.^36^. The adsorption behavior of APA on the Pd_1_-terminated PdGa{111} surfaces is in stark contrast to that on A:Pd_3_, as the APA molecules barely agglomerate even at temperatures up to 415 K (Fig. 3b and Fig. S5). The most frequently observed APA structures are shown in the STM images in Fig. 3c–f. The sickle-like molecular structures in Fig. 3c, e strongly resemble the terminal phenylpropionic acid group of the ex situ synthesized 1,4-triazoles adsorbed on PdGa:A(111)Pd_1_ in Fig. S6. These two structures are attributed to pristine APA molecules, whereas their azide group only exhibits a very weak STM signal like on the less corrugated Ag(111)^37^. The coverage of the molecular structures in Fig. 3c, e relative to all APA derivatives on the Pd_1_-terminated PdGa{111} surfaces amounts to around 30–35%, upon deposition and after annealing to 415 K (Fig. S5), which is in good agreement with the maximum amount of pristine APA molecules estimated from XPS investigations (Fig. S4 and Table S5). The molecular structures in Fig. 3d, f are APA molecules with decomposed azide group. On Pd_1_-terminated PdGa{111} surfaces, part of the APA propionic acid groups are deprotonated (Fig. S2b and Table S2), but no signs of their detachment from APA molecules could be identified, neither with XPS nor STM.

**Fig. 4:**
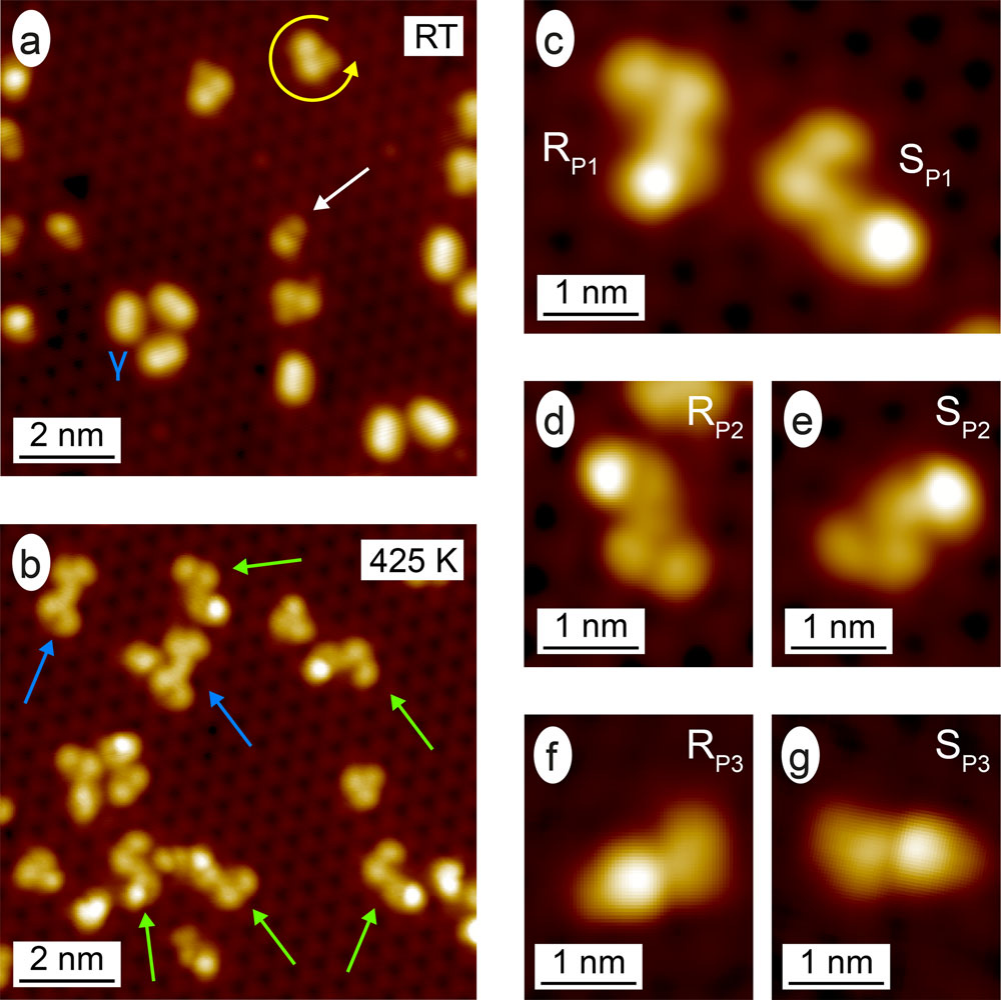
Enantioselective azide–alkyne Huisgen cycloaddition on the Pd_1_-terminated PdGa{111} surfaces. STM images (*V*_B_ = −200 mV; *I*_T_ = 0.05 nA) of **a** co-deposited APA (white arrow) and 9-EP (yellow arrow and γ-states labeled) molecules on PdGa:B($\bar 1\bar 1\bar 1$)Pd_1_, and after subsequent annealing to **b** 425 K. In **b**, the 9-EP dimers are pointed out with blue arrows, and the 1,4-triazoles with green ones. The different 1,4-triazoles are shown in detail in the high-resolution STM images (*V*_B_ = −200 mV; *I*_T_ = 0.2 nA) in **c**–**g**. **d** and **g** have been rotated to highlight the relation of the shown 1,4-triazole to the one in **e** and **f**, respectively.

After having clarified that around 30% of the APA monomers remain structurally intact up to 415 K on the Pd_1_-terminated PdGa{111} surfaces, APA was co-deposited with 9-EP at RT (Fig. 4). As shown in Fig. 4a, both 9-EP and APA mainly appear as non-interacting monomers. The pronounced depressions on this surface represent Pd vacancies in the Pd_1_-termination^26^ and the apparent trimer is composed of individual 9-EP molecules adsorbed on such a vacancy^36^. 9-EP molecules appear with a relative abundance between 40 and 60% and exhibit an ee > 90%. Due to the lack of significant intermolecular interactions, also APA molecules occupy the same adsorption sites as in the case when they are adsorbed without 9-EP (cf. Fig. 3).

The cycloaddition between APA and 9-EP can be triggered thermally on Pd_1_-terminated PdGa{111} by annealing the sample with co-deposited molecules at 425 K. This gives rise to the observation of several new covalently coupled molecular structures (Fig. 4b) in high abundance (Figs. S9 and S10 and Supplementary Note 2). About 5% of the new molecular structures are previously reported 9-EP dimers^36^ (blue arrow in Fig. 4b), whereas 5% consist of one 9-EP molecule and the phenylpropionic acid moiety of APA, but are too small to be intact triazoles (Fig. S11). The vast majority (90%, green arrows Fig. 4b) of these molecular structures exhibit one of the six different STM appearances presented in Fig. 4c–g. Their STM signature is mainly planar in the vicinity of the coupling site, which excludes them to be 1,5-triazoles, as they would exhibit highly non-planar STM signatures according to DFT calculations done with CP2K^38^ within the AiiDalab platform^39^ (Fig. S12).

To check whether the six molecular structures in Fig. 4c–g are on-surface synthesized 1,4-triazoles, we deposited ex situ synthesized 1,4-triazoles on A:Pd_1_ held at 170 and 300 K. When imaged with STM, the ex situ synthesized 1,4-triazoles appear in three inequivalent adsorption configurations (Fig. S6). These 1,4-triazole molecules strongly resemble the reaction products formed between APA and 9-EP on the Pd_1_-terminated PdGa{111} in terms of shape, dimensions, and adsorption geometry (Fig. S7), but differ in the intensity distribution. However, upon annealing the ex situ synthesized 1,4-triazoles to the reaction temperature of the on-surface synthesis, we observe molecular structures that are identical to those formed between APA and 9-EP on the Pd_1_-terminated PdGa{111} surfaces (Fig. S8). This implies that the vast majority of the heterocoupled reaction products are indeed 1,4-triazoles, but with deprotonated carboxylic acid. Hence, the azide–alkyne Huisgen cycloaddition proceeds regioselectively on Pd_1_-terminated PdGa{111} surfaces.

### Asymmetric azide–alkyne Huisgen cycloaddition on Pd_1_-terminated PdGa{111} surfaces

It becomes clear that the two molecules in Fig. 4c, the two in Fig. 4d, e and the ones in Fig. 4f, g are mirror images of each another, thus they represent complementary enantiomers, which will be discussed later in view of enantioselectivity. The distinctive feature in the STM signatures of the R and S enantiomers in all three cases is the arrangement of the phenylpropionic acid with respect to the phenanthrene moiety. If the phenylpropionic acid unit is perpendicular (almost parallel) to the phenanthrene moiety, the 1,4-triazoles appear L-shaped (Z-shaped) (Fig. 4c–e, the triazoles are denoted P1 and P2, respectively) or I-shaped (P3) for an intermediate orientation (Fig. 4f, g). The atomic resolution of the substrate in the STM images combined with the structure overlay allows to determine the adsorption configuration of these reaction products (Fig. S13b, e, h), whereas the enantiospecific arrangement of the propionic acid moiety remains ambiguous. The relative abundance of the three product structures P1–P3 are summarized in Table 1 for the two enantiomeric forms R and S on the Pd_1_-terminated surfaces (the products are correspondingly labeled R_P1_, S_P1_, R_P2_,…, S_P3_). Regarding the arrangement of the triazole with respect to the phenanthrene moiety in the 1,4-triazoles, P2 and P3 belong to the same diastereomer. Hence the reaction proceeds with a diastereomeric excess ${\mathrm{{de}}} = 100\% \ast \frac{{{\mathrm{P}}2 + {\mathrm{P}}3 - P1}}{{{\mathrm{{P2 + P3 + P1}}}}}$ of up to 50%. Considering all 1,4-triazoles, the 9-EP moiety appear as racemic mixture, which implies that at the reaction temperature of 425 K, the chiral recognition of 9-EP by the Pd_1_-terminated PdGa{111} surfaces has become insignificant. Unlike the Z-shaped products P2, which do not experience any chirality transfer, enantioselectivity is clearly expressed for the L- and I-shaped products P1 and P3, where we find ratios between the different enantiomorphs of up to 4:1 and 5:1, respectively. This enantioselective behavior is supported by deposited ex situ synthesized 1,4-triazoles pointing towards the same preferential arrangement (cf. Table S6). The opposite ratio between the different P1 and P3 enantiomers for Pd_1_-terminated PdGa{111} surfaces of the A and B PdGa crystal enantiomorph strongly corroborates the conclusion of their enantioselective recognition towards the 1,4-triazoles.

**Table 1:** Quantification of the reaction products.

Sample	1,4-Triazoles						9-EP dimer	Others
	R_P1_	S_P1_	R_P2_	S_P2_	R_P3_	S_P3_		
A:Pd_1_	5 ± 1%	21 ± 2%	13 ± 2%	14 ± 2%	30 ± 2%	6 ± 1%	6 ± 1%	5 ± 1%
B:Pd_1_	18 ± 2%	3 ± 1%	10 ± 2%	10 ± 2%	12 ± 2%	36 ± 2%	5 ± 1%	6 ± 1%

Abundancies of the identified coupled molecular species on the PdGa:A(111)Pd_1_ and PdGa:B($\bar 1\bar 1\bar 1$)Pd_1_ surfaces. The reaction products P1 and P3 clearly occur enantioselectively, while the products P2 appear in a racemic mixture.

Compared to investigations of the azide–alkyne Huisgen cycloaddition on Cu(111)^34^, where the reaction yield is reported to be of the order of 2–10%, the formation of 1,4-triazoles on the Pd_1_-terminated PdGa{111} surfaces reveals a much higher yield of up to 58%. It seems as if this yield depends mainly on the availability of intact APA molecules as the yield increases with increasing ratio between APA/9-EP (Table S7), which is expected in light of the decomposition of the azid group of APA molecules upon deposition on the Pd_1_-terminated PdGa{111} surfaces.

## Conclusion

In summary, we have shown that the azide–alkyne Huisgen cycloaddition between pristine 9-EP and APA occurs regio- and enantioselectively on the Pd_1_-terminated PdGa{111} surfaces, i.e., forming exclusively 1,4-triazoles with an exceptionally high enantiospecifity of up to 5:1. In view of the d-band model, the Pd_1_-terminated PdGa{111} surfaces behave copper-like. On the other hand, the same reaction does not proceed on the Pd_3_-terminated PdGa{111} surfaces, even though these PdGa{111} surfaces exhibit very similar electronic d-band structure. We therefore conclude that the vastly dissimilar catalytic properties of the Pd_1_- and Pd_3_-terminated PdGa{111} surfaces for the azide–alkyne Huisgen cycloaddition primarily arise from differences in the atomic arrangement in their terminating layers, i.e., the ensemble effect.

## Methods

### Synthesis of 3-(4-(4-(phenanthren-9-yl)-1H-1,2,3-triazol-1-yl)phenyl)propionic acid

Chemicals and solvents were purchased from commercial sources (Merck, VWR) and were used without further purification. Reactions were carried out under argon atmosphere. Column chromatography was performed using silica gel (pore size 40–63 μm, Normasil 60^®^ from VWR chemicals).

9-Ethynylphenanthrene (75 mg, 0.37 mmol), 3-(4-azidophenyl)propinoic acid (57 mg, 0.30 mmol), tris((1-benzyl-1*H*-1,2,3-triazol-4-yl)methyl)amine (11 mg, 0.02 mmol), copper(II) sulfate (7 mg, 0.04 mmol), and sodium ascorbate (18 mg, 0.09 mmol) were dissolved in 6 ml *N*,*N*-dimethylformamide and 2 ml water. The reaction mixture was stirred for 6 h at room temperature. Additional water was added and the product was extracted with ethyl acetate. The organic fractions were washed with water and brine and dried with anhydrous sodium sulfate. The crude product was purified by flash column chromatography with silica gel and a solvent mixture of dichloromethane/ethyl acetate/acetic acid. The triazole was precipitated from dichloromethane/*n*-heptane and isolated as a white crystalline solid (85 mg, 0.22 mmol, 72%).

### Nuclear magnetic resonance

^1^H and ^13^C NMR data were recorded at 400.2 and 100.6 MHz using a 5 mm CryoProbe™ Prodigy probe equipped with *z*-gradient on a Bruker Avance III 400 NMR spectrometer (Bruker Biospin AG, Fällanden, Switzerland). 1D ^1^H and ^13^C NMR experiments as well as 2D-correlated ^1^H-^13^C HSQC, ^1^H-^13^C HMBC, and ^1^H-^1^H DQF-COSY experiments were performed at 298 K using the Bruker standard pulse programs and parameter sets applying 90° pulse lengths of 11.4 µs (^1^H) and 10.0 µs (^13^C). Chemical shifts (*δ* in ppm) were calibrated to residual solvent peaks of CDCl_3_ at *δ* = 7.26 and 77.0 ppm. Coupling constants *J* are reported in Hz and ^1^H NMR multiplicities are described as s = singlet, d = doublet, t = triplet, m = multiplet, and dd = doublet of doublet. High-resolution mass spectrometry was recorded on a Bruker Daltonics maXis II ESI-QTOF mass spectrometer in positive mode.

^1^H NMR (CDCl_3_, 400.2 MHz): *δ* 8.73 (d, *J* = 8.2 Hz, 1H), 8.45 (dd, *J* = 8.2, 1.0 Hz, 1H), 8.28 (s, 1H), 8.08 (s, 1H), 7.94 (d, *J* = 7.1 Hz, 1H), 7.82 (d, *J* = 8.5 Hz, 2H), 7.75–7.68 (m, 2H), 7.68–7.61 (m, 2H), 7.45 (d, *J* = 8.5 Hz, 2H), 3.09 (t, *J* = 7.6 Hz, 2H), 2.78 (t, *J* = 7.6 Hz, 2H).

^13^C NMR (CDCl_3_, 100.6 MHz): *δ* 174.4, 147.5, 141.2, 135.5, 131.3, 130.7, 130.6, 130.3, 130.1, 129.8, 129.0, 128.8, 128.6, 127.2, 127.0, 127.0, 126.8, 126.2, 123.1, 122.6, 120.9, 34.6, 30.2.

HR-MS (pos. ESI): *m*/*z* for [C_25_H_20_N_3_O_2_^+^]; calculated 394.1550, found 394.1549.

### Sample preparation and experiments

All experiments and sample preparations were performed under UHV conditions with a base pressure below 2 × 10^−10^ mbar.

The sample was prepared by repeated sputtering and annealing cycles (sputtering: 20 min, Ar^+^, 1 keV; annealing: 20 min at 870 K). Both 9-ethynylphenanthrene (9-EP; 97%) and 3-(4-azidophenyl)propionic acid (APA; ≥97%) were purchased from Sigma-Aldrich and were used without further purification. 9-EP and APA were deposited by exposing the clean sample surface to the molecules held at 300 K in a pumped glass tube separated by a gate valve from the preparation chamber to prevent contamination. 9-EP and APA were pumped via individual connections with the same turbo pump to minimize the risk of cross-contamination.

3-(4-(4-(phenanthrene-9-yl)-1*H*-1,2,3-triazol-1-yl)phenyl)propionic acid, further referred to as ex situ synthesized 1,4-triazole, was deposited on the clean sample surface held at 300 K from a Knudsen cell at 490 K.

STM images were acquired with a commercial low temperature STM from Scienta Omicron operated at 5 K, if not mentioned differently, in constant-current mode.

All XPS data were recorded in normal emission configuration. XPS experiments shown in Fig. S2 were performed in-house and at room temperature using a monochromatized Al *K*α source and a Scienta R3000 display analyzer. XPS spectra shown in Figs. S3 and S4 were recorded at the X03DA beamline (PEARL endstation)^40^ of the SLS synchrotron radiation facility (PSI, Villigen, Switzerland) using linearly polarized radiation with a photon energy of 760 eV and a Scienta R4000 hemispherical electron analyzer equipped with a multichannel plate detector with the sample held at 180 K.

### DFT calculations

The DFT calculations were performed using the CP2K code^38^ on the AiiDAlab platform^39^. The electronic states were expanded with a TZV2P Gaussian basis set^41^ for C, H, N, and O species and a DZVP basis set for Pd and Ga species. A cutoff of 1200 Ry was used for the plane wave basis set. We used Norm Conserving Goedecker-Teter-Hutter pseudopotentials^42^ and the PBE exchange-correlation functional^43^ with the D3 dispersion corrections proposed by Grimme^44^.

The Pd_1_ surface was modeled by a supercell of 34.75 × 36.11 Å^2^ corresponding to 30 surface units. To obtain the equilibrium geometries, we kept the atomic positions of the bottom seven layers of the slab fixed to the bulk positions, and all other atoms were relaxed until forces were lower than 0.005 eV/Å. To obtain simulated STM images^45^ within the Tersoff-Hamann approximation^46,47^ we extrapolated the electronic orbitals to the vacuum region following the approach outlined by Tersoff^47^.

## Supplementary information

Supplementary Information

## Data Availability

The data supporting the findings of this study are included in the main text and in the Supplementary Information files, and available on archive.materialscloud.org (DOI: 10.24435/materialscloud:tx-8g).
