# Supplementary material for: Asymmetric azide-alkyne Huisgen cycloaddition on chiral metal surfaces
Source: Commun Chem. 2021 Apr 12;4:51. doi: 10.1038/s42004-021-00488-0 (PMC9814088; doi:10.1038/s42004-021-00488-0)
Supplement: Supplementary file 1 — Supplementary Information [file 42004_2021_488_MOESM1_ESM.pdf]

Supporting information for

Asymmetric azide-alkyne Huisgen cycloaddition on chiral metal surfaces

Samuel Stolz[1,2], Michael Bauer[3], Carlo A. Pignedoli[1], Nils Krane[1], Max Bommert[1], Elia Turco[1], Nicolo Bassi[1], Amogh Kinikar[1], Néstor Merino-Dìez[1], Roland Hany[3], Harald Brune[2], Oliver Gröning[1], and Roland Widmer[1,*]

[1]*Empa, Swiss Federal Laboratories for Materials Science and Technology, nanotech@surfaces Laboratory, 8600 Dübendorf, Switzerland*
[2]*Institute of Physics, École Polytechnique Fédérale de Lausanne, Laboratory of Nanostructures at Surfaces, CH-1015 Lausanne, Switzerland*
[3]*Empa, Swiss Federal Laboratories for Materials Science and Technology, Laboratory for Functional Polymers, 8600 Dübendorf, Switzerland*

Table of Contents

Deposition of ex-situ synthesized 1,4-triazoles on PdGa:A($\bar{1}\bar{1}\bar{1}$)Pd_3 2

XPS of APA on PdGa:A($\bar{1}\bar{1}\bar{1}$)Pd_3, PdGa:A(111)Pd_1 and Cu(111) 3

Temperature evolution of APA on PdGa:B($\bar{1}\bar{1}\bar{1}$)Pd_1 8

Ex-situ synthesized 1,4-triazoles deposited on PdGa:A(111)Pd_1 9

Abundance of reaction products 11

Side products of the azide-alkyne Huisgen cycloaddition on Pd_1-terminated PdGa{111} surfaces 13

DFT simulations of 1,4- and 1,5-triazoles 14

Adsorption configurations of the 1,4-triazoles 15

Determination of reaction yield 16

Supplementary References 17

Deposition of ex-situ synthesized 1,4-triazoles on PdGa:A($\overline{1}\overline{1}\overline{1}$)$Pd_3$

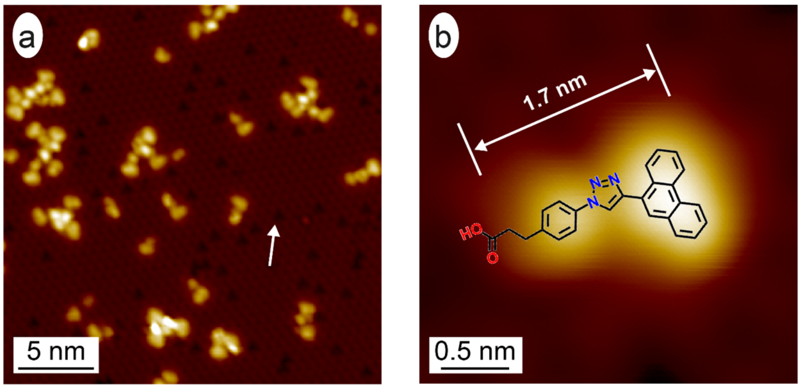

Figure S1

STM images of ex-situ synthesized 1,4-triazoles deposited on the PdGa:A($\overline{1}\overline{1}\overline{1}$)$Pd_3$ surface at 300 K (**a** $V_B = 50\ mV$; $I_T = 20\ pA$; **b** $V_B = 50\ mV$; $I_T = 50\ pA$).

XPS of APA on PdGa:A($\bar{1}\bar{1}\bar{1}$)Pd$_3$, PdGa:A(111)Pd$_1$ and Cu(111)

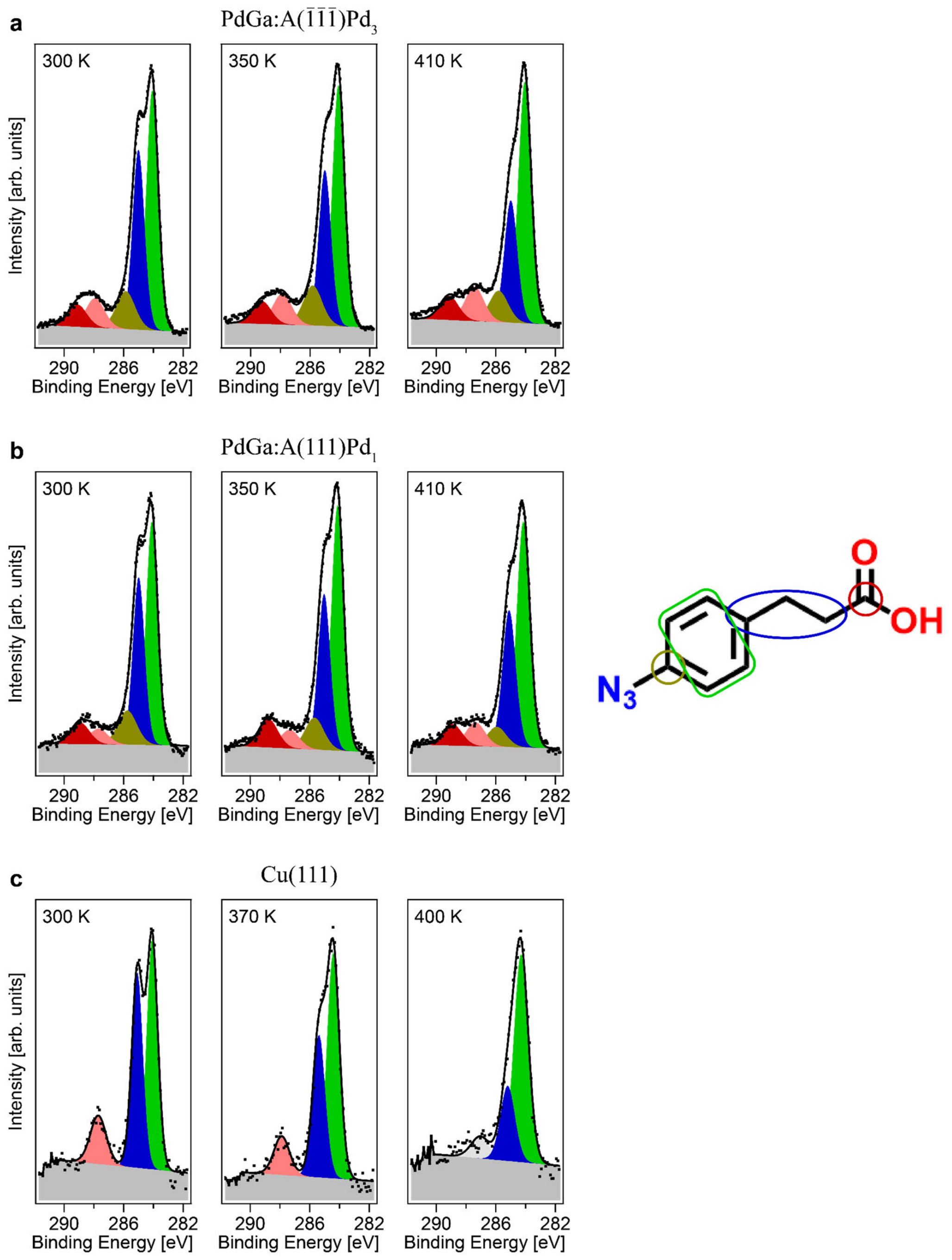

Figure S2

C 1s XPS core level signal of APA on **a** PdGa:A($\bar{1}\bar{1}\bar{1}$)Pd$_3$, **b** PdGa:A(111)Pd$_1$, and **c** Cu(111) after deposition at 300 K (left) and after annealing to the temperatures indicated in the top left corner of each XPS spectrum. On the right a sketch of the APA molecule is displayed with the carbon atoms contributing to each part of the XPS signal is colored according to the colors of the individual components in the XPS spectra. The carboxylic acid contributes two components to the XPS spectra, one corresponding to its inert state (dark red), and the other to the formation of carboxylate (bright red).

PdGa:A($\bar{1}\bar{1}\bar{1}$)Pd$_3$

	300 K		350 K		410 K	
	BE [eV]	Weighting	BE [eV]	Weighting	BE [eV]	Weighting
COOH	289.2	6.3	289.3	6.6	289.2	7.1
COO-	288	8.3	287.9	8.8	287.5	10.2
C-N	285.8	10.9	285.9	11.4	285.9	9.3
"C3"	285.1	32.8	285.1	28.8	285.1	24.7
"C4"	284.2	43.7	284.2	44.5	284.2	48.8

Table S1

Fitting parameters for the XPS spectra of APA on PdGa:A($\bar{1}\bar{1}\bar{1}$)Pd$_3$ for the temperatures indicated in Figure S2a. For each temperature, the binding energy (BE) and weight of all fit components depicted in the molecular structure in Figure S2 are given.

PdGa:A(111)Pd$_1$

	300 K		350 K		410 K	
	BE [eV]	Weighting	BE [eV]	Weighting	BE [eV]	Weighting
COOH	289.0	7.0	288.9	8.5	289.0	6.7
COO-	287.8	5.1	287.4	5.5	287.5	7.5
C-N	285.9	11.0	285.8	9.4	286.0	9.3
"C3"	285.1	33.0	285.1	29.7	285.2	30.0
"C4"	284.2	44.0	284.2	46.8	284.3	49.5

Table S2

Fitting parameters for the XPS spectra of APA on PdGa:A(111)Pd$_1$ for the temperatures indicated in Figure S2b. For each temperature, the binding energy (BE) and weight of all fit components depicted in the molecular structure in Figure S2 are given.

Cu(111)

	300 K		370 K		400 K	
	BE [eV]	Weighting	BE [eV]	Weighting	BE [eV]	Weighting
COOH	-	0	-	0	-	0
COO-	287.9	14.1	288	11.6	-	0
C-N	-	0	-	0	-	0
Unknown	-	0	-	0	287.2	8.9
"C3"	285.2	39.5	285.5	34.2	285.4	24.2
"C4"	284.2	46.4	284.6	54.1	284.5	66.9

Table S3

Fitting parameters for the XPS spectra of APA on Cu(111) for the temperatures indicated in Figure S2c. For each temperature, the binding energy (BE) and weight of all fit components depicted in the molecular structure in Figure S2 are given.

Supplementary Note 1

For reactivity investigations, we are mainly interested in the two - out of the overall 9 - C atoms of the APA molecule (cf. Figure S2) that are either part of propionic acid, or directly connected to the azide group. Each of these two C atoms in APA give rise to a component in the C 1s core level signal with a chemical shift to higher binding energy with respect to the remaining 7 C atoms of the backbone with an expected weight of 11%.

Because no information is found on the chemical shift in the XPS of the C 1s core level induced by azide functional groups,[1] we identify the contribution of the C atom attached to the azide group to the C 1s XPS signal by referring to Ref. [2], where an XPS investigation of a molecule with a diazo group was performed. The carbon atom attached

to the diazo group has been reported to give rise to a C 1s component shifted by about 1 eV towards higher binding energy (but still a lower shift than for carbon in carboxylic groups) with respect to the C 1s component of aromatic carbon atoms. We therefore assign the components denoted with C-N in Table S1-Table S3 to originate from the carbon atom attached to the azide group.

For APA on the PdGa{111} surfaces this C-N XPS component is discernible with an amount of 6-11% (depending on the fitting constraints), whereas it is lacking for APA on Cu(111). Based on the N 1s core level XPS signal on Cu(111), we estimate that only 25% of the expected N 1s signal for inert APA is detected, which indicates a large quantity of the azide groups to be detached from APA, in agreement with previous reports on Cu(111).[1]

Contrary to azide functional groups, detailed XPS investigations on carboxylic acids have been reported on Au(111), Ag(100), Ag(111), Cu(100), Cu(110) and Cu(111) surfaces.[3–9] In XPS, this functional group can be identified due to the large chemical shift in the C 1s core level signal towards higher binding energy compared to aromatic carbon, resulting in a C 1s core level component at about 289.3 eV. Upon deprotonation of the COOH group, a new C 1s core level component emerges at around 288.1 eV.[8,9]

Applying these results to our XPS experiments in Figure S2 and Table S1-Table S3above, we find that the carboxylic acid of the APA molecules form a carboxylate upon deposition at 300 K on the Cu(111) surface. Contrary to the PdGa{111} surfaces where more than 40% (Pd_3-terminated PdGa{111}) or even close to 60% (Pd_1-terminated PdGa{111}) of all carboxylic acids remain protonated. This ratio remains stable even upon annealing to 350 K. Moreover, while on Cu(111), the XPS signal originating from the carboxylic acid group vanishes upon annealing to 400 K, it persists on PdGa{111} up to temperatures of 410 K with a weighting of maximal 17%.

We also determined the experimental C 1s/N 1s XPS intensity ratio for APA and ex-situ synthesized 1,4-triazoles on PdGa:A(111)Pd_1 and PdGa:A($\bar{1}\bar{1}\bar{1}$)Pd_3 to estimate the upper limit of relative coverage of pristine APA molecules and to determine their thermal stability.

As seen in Table S4 and Table S5, the determined C 1s/N 1s XPS intensity ratio is about a factor of 3 for PdGa:A($\bar{1}\bar{1}\bar{1}$)Pd_3 and 3.5 for PdGa:A(111)Pd_1 larger than expected - i.e., 7.0 and 7.9 instead of 2.2 - if we assumed all ex-situ synthesized 1,4-triazoles to be pristine. This implies that at most around 30% of all APA molecules exhibit an intact azide group required for the azide-alkyne Huisgen cycloaddition – which is similar to the percentage that has been reported for 4-azidobiphenyl on Cu(111).[1] Nevertheless, the pristine APA molecules co-deposited with 9-EP are thermally stable on PdGa:A(111)Pd_1, at least to the reaction temperature because the C 1s/N 1s XPS intensity ratio is roughly the same for APA and 9-EP co-deposited at 300 K and after annealing to the reaction temperature.

To obtain a reasonable core level signal in XPS, we had to prepare samples with high coverage of 9-EP and APA molecules as shown in Figure S4b. Therefore, the amount of reaction products is low due to the reduced mobility of the molecules at such coverage.

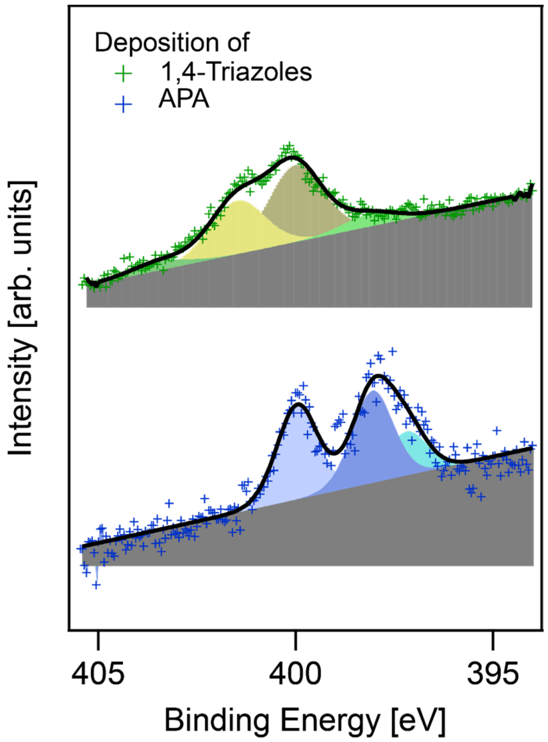

Figure S3

N 1s XPS signal of ex-situ synthesized 1,4-triazoles (upper curve) and APA (lower curve) deposited at 300 K on PdGa:A(111)Pd_3. The markers represent the experimental XPS data, the solid grey areas the background, the colored areas the individual N 1s components and the solid overall fitted intensity.

	APA	1,4-triazoles
C1s/N1s	7.0 (2.2)	6.4

Table S4

Experimental C 1s/N 1s XPS intensity ratios for APA and ex-situ synthesized 1,4-triazoles adsorbed on the PdGa:A($\bar{1}\bar{1}\bar{1}$)Pd_3 surface. The number in brackets for the case of APA molecules corresponds to the expected N 1s/C 1s XPS intensity ratio under the assumption that all 1,4-triazoles remain pristine upon deposition.

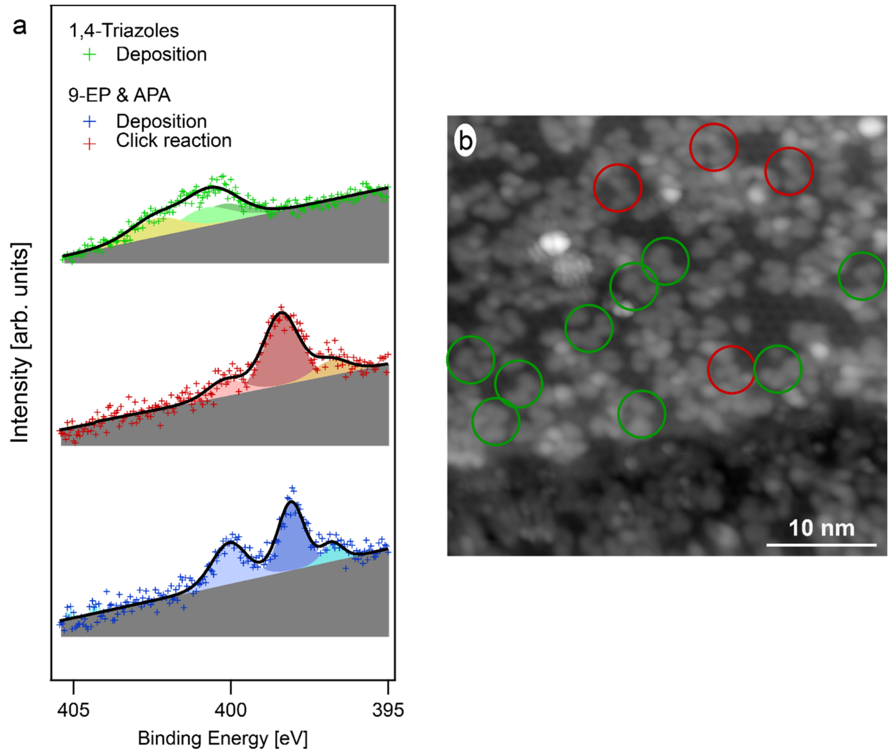

Figure S4

a N 1s signal of ex-situ synthesized 1,4-triazoles deposited at 300 K, APA co-adsorbed with 9-EP at 300 K and after triggering the reaction on PdGa:A(111)Pd_1. The markers represent the experimental XPS data, the solid black areas the background, the colored areas the individual N 1s components and the solid overall fitted intensity. **b** STM image ($V_B = -500\ mV$; $I_T = 10\ pA$) illustrating the coverage of co-deposited 9-EP and APA after initializing the click reaction. Marked in red are the reaction products and in green 9-EP dimers.

	APA	1,4-triazoles	APA & 9-EP	APA & 9-EP (reaction)
C1s/N1s	7.9 (2.2)	6.0	13.9	12.9

Table S5

Experimental C 1s/N 1s XPS intensity ratios for APA, ex-situ synthesized 1,4-triazoles, and APA and 9-EP adsorbed on the PdGa:A(111)Pd_1 surface. The number in brackets for the case of APA molecules corresponds to the expected N 1s/C 1s XPS intensity ratio, when assuming all 1,4-triazoles to be pristine.

Temperature evolution of APA on PdGa:B($\bar{1}\bar{1}\bar{1}$)Pd$_1$

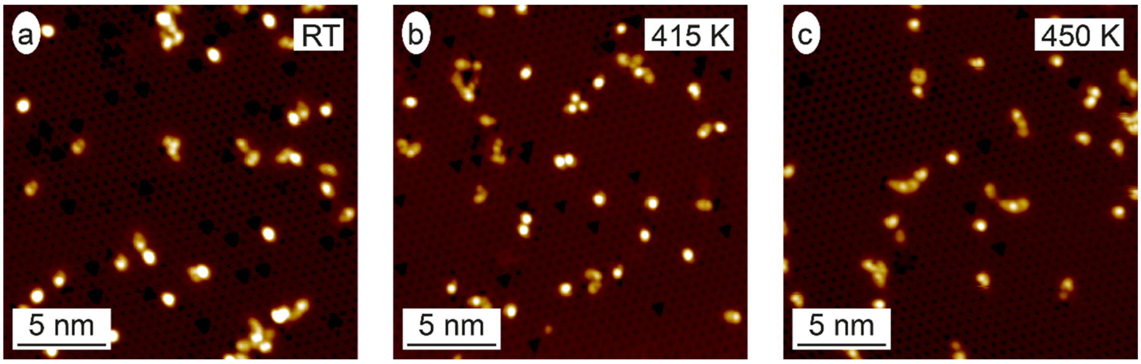

Figure S5
STM images ($V_B = 200\,mV$; $I_T = 200\,pA$) of APA molecules after **a** room temperature deposition, subsequent annealing to **b** 415 K and **c** 450 K.

From room temperature to 415 K, APA appears mostly isolated and in the four configurations shown in Figure 3c-f of the main text. Only upon annealing to 450 K, agglomeration becomes more frequent and the configurations shown in Figure 3c,e of the main text completely disappear.

Ex-situ synthesized 1,4-triazoles deposited on PdGa:A(111)Pd$_1$

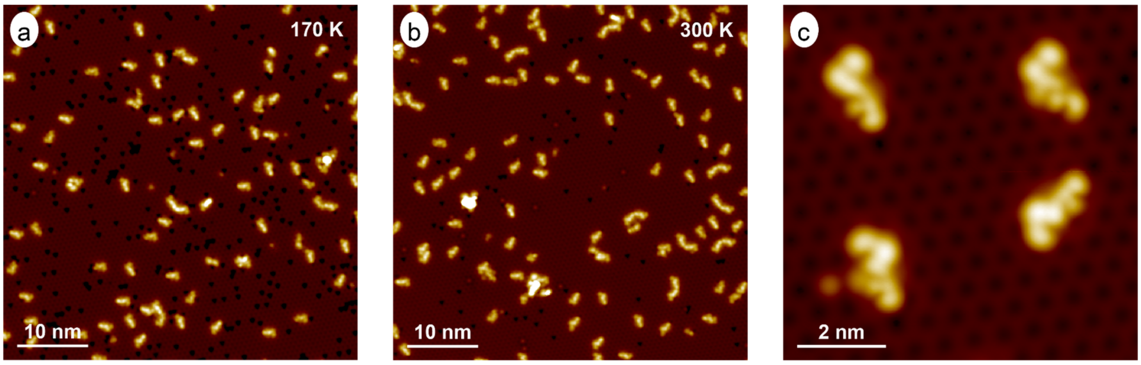

Figure S6

STM images ($V_B = 1000\ mV$; $I_T = 20\ pA$) of the ex-situ synthesized 1,4-triazoles deposited at **a** 170 K and **b** 300 K. In **c**, the STM signatures ($V_B = 50\ mV$; $I_T = 100\ pA$) of the three observed adsorption configurations of deposited 1,4-triazoles are depicted.

Sample	ex-situ synthesized 1,4-triazoles		
	S_{P1*} 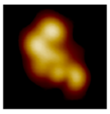	S_{P2*} 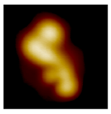	R_{P2*} 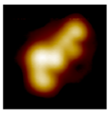
A:Pd$_1$	9 ± 2%	39 ± 3%	52 ± 4%

Table S6

Relative abundance of the adsorption configurations of ex-situ synthesized 1,4-triazoles on PdGa:A(111)Pd$_1$ at 300 K.

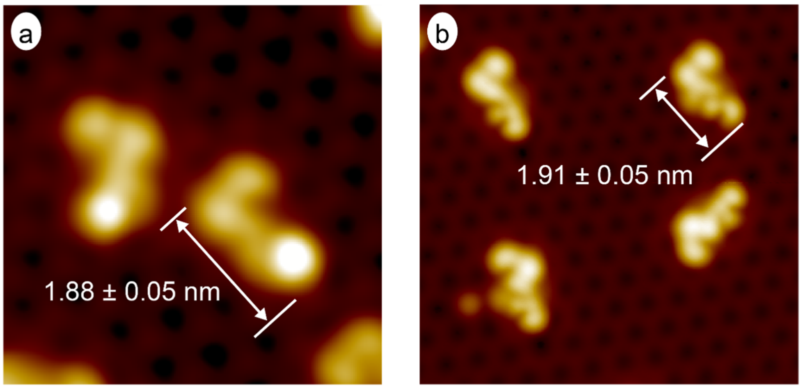

Figure S7

STM images of **a** the on-surface synthesized reaction products between 9-EP and APA and **b** ex-situ synthesized 1,4-triazoles on Pd$_1$-terminated PdGa{111} surfaces.

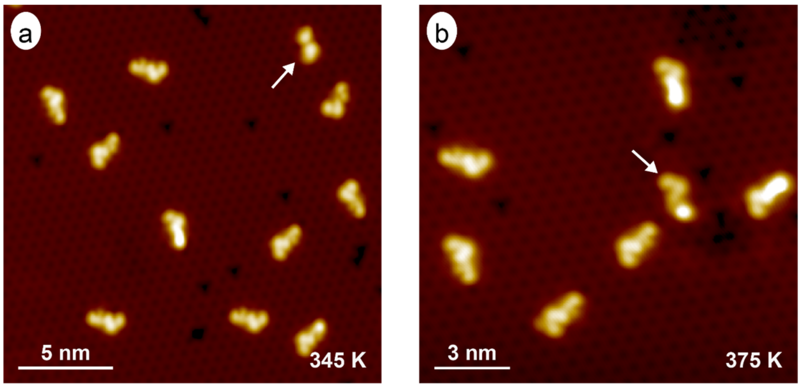

Figure S8
STM images of the ex-situ synthesized 1,4-triazoles after annealing to **a** 345 K ($V_B = 50\ mV$; $I_T = 100\ pA$) and **b** 375 K ($V_B = 50\ mV$; $I_T = 20\ pA$). The white arrows indicate molecular structures that are identical to the ones obtained by on-surface synthesis between APA and 9-EP on the Pd_1-terminated PdGa$\{111\}$ surfaces.

Abundance of reaction products

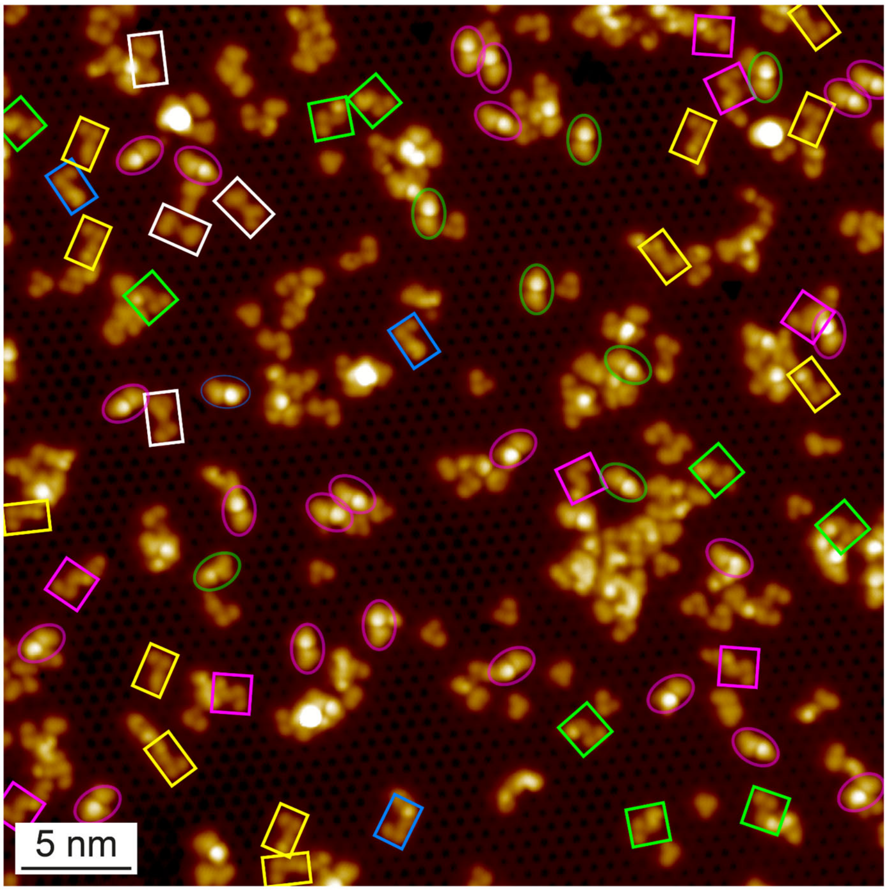

Figure S9
40 x 40 nm^2 STM image after thermally triggering the azide-alkyne Huisgen cycloaddition on the PdGa:B($\bar{1}\bar{1}\bar{1}$)Pd_1 ($V_B = 100\ mV$; $I_T = 100\ pA$) by annealing to 425 K. All 1,4-triazoles and 9-EP dimers are highlighted by colored rectangles or circle (see text).

Supplementary Note 2

The colors of the rectangles and circles in Figure S9 and Figure S10 indicate the same reaction product. Yellow rectangles correspond to R_{P1}, the blue ones to S_{P1}, the green ones to R_{P2}, the pink ones to S_{P2} and the white ones to 9-EP dimers. The red circles highlight the S_{P3} and the green circles the R_{P3} 1,4-triazole, respectively. For the statistical analysis, no structures belonging to larger molecule agglomerations are considered, as can be seen from Figure S9 and Figure S10. As a consequence, the determined yield of reaction products given in Table S7 potentially underestimates the actual yield.

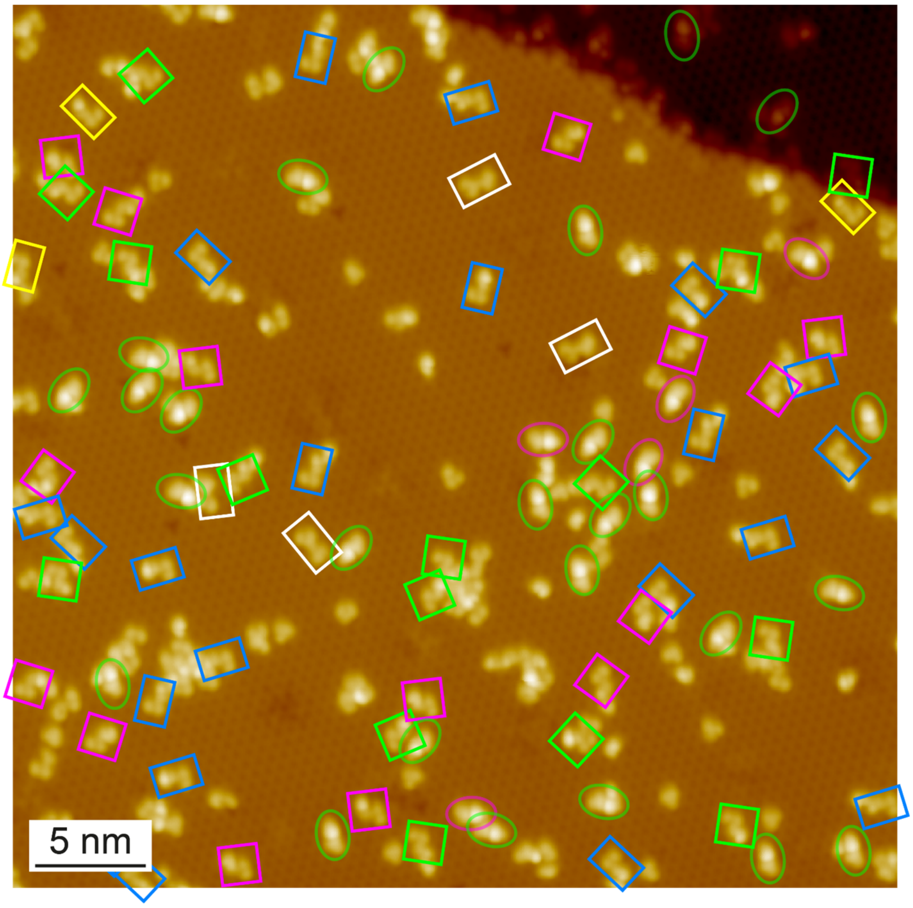

Figure S10

40 x 40 nm^2 STM image after thermally triggering the azide-alkyne Huisgen cycloaddition on the PdGa:A(111)Pd$_1$ ($V_B = 100\ mV$; $I_T = 100\ pA$) by annealing to 425 K. All 1,4-triazoles and 9-EP dimers are highlighted by colored rectangles or circles (see text).

Side products of the azide-alkyne Huisgen cycloaddition on Pd$_1$-terminated PdGa{111} surfaces

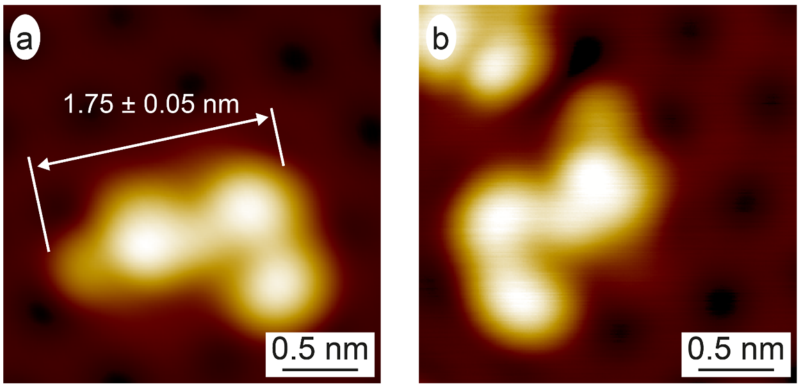

Figure S11

STM images (**a** $V_B = 50$ mV, $I_T = 250$ pA; **b** $V_B = 10$ mV, $I_T = 5$ pA) of side products of the azide-alkyne Huisgen cycloaddition on Pd$_1$-terminated PdGa{111} surfaces. These side products are probably 9-EP coupled to the phenylpropionic acid originating from azide decomposition of APA molecules.

DFT simulations of 1,4- and 1,5-triazoles

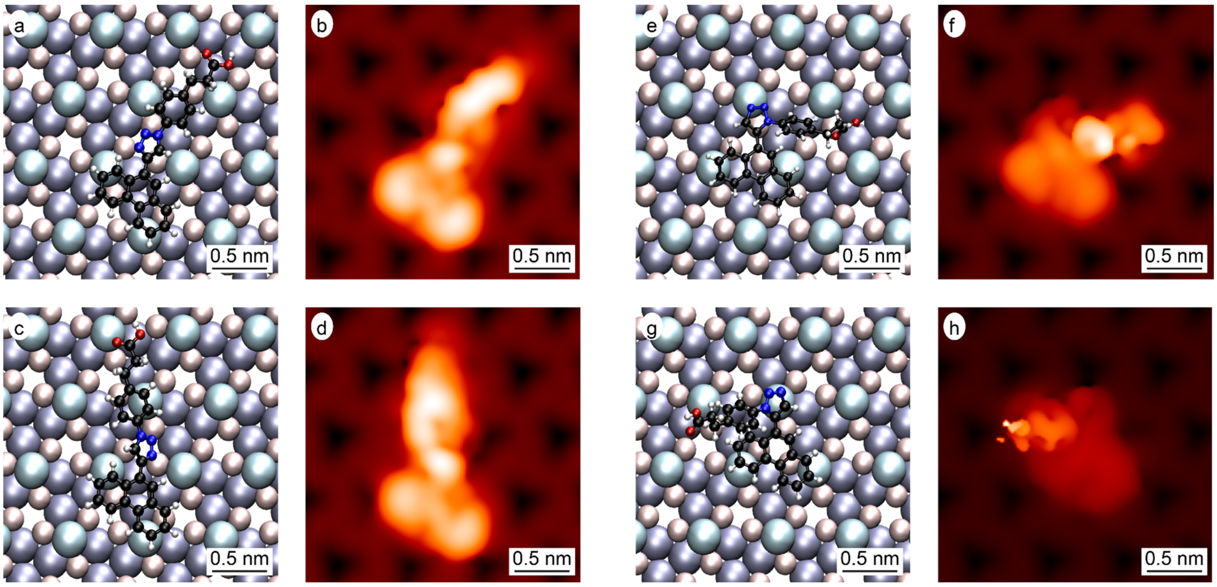

Figure S12
DFT-optimized adsorption configurations (**a,c,e,g**) with the corresponding DFT-simulated STM signature (**b,d,f,h**) for **a-d** 1,4-triazoles and **e-h** 1,5-triazole diastereomers. The two diastereomers of the 1,4- and 1,5-triazoles exhibit the same adsorption configuration in their phenanthrene unit, while the remaining atoms are rotated by 180° around the bond connecting the phenanthrene with the triazole unit.

Adsorption configurations of the 1,4-triazoles

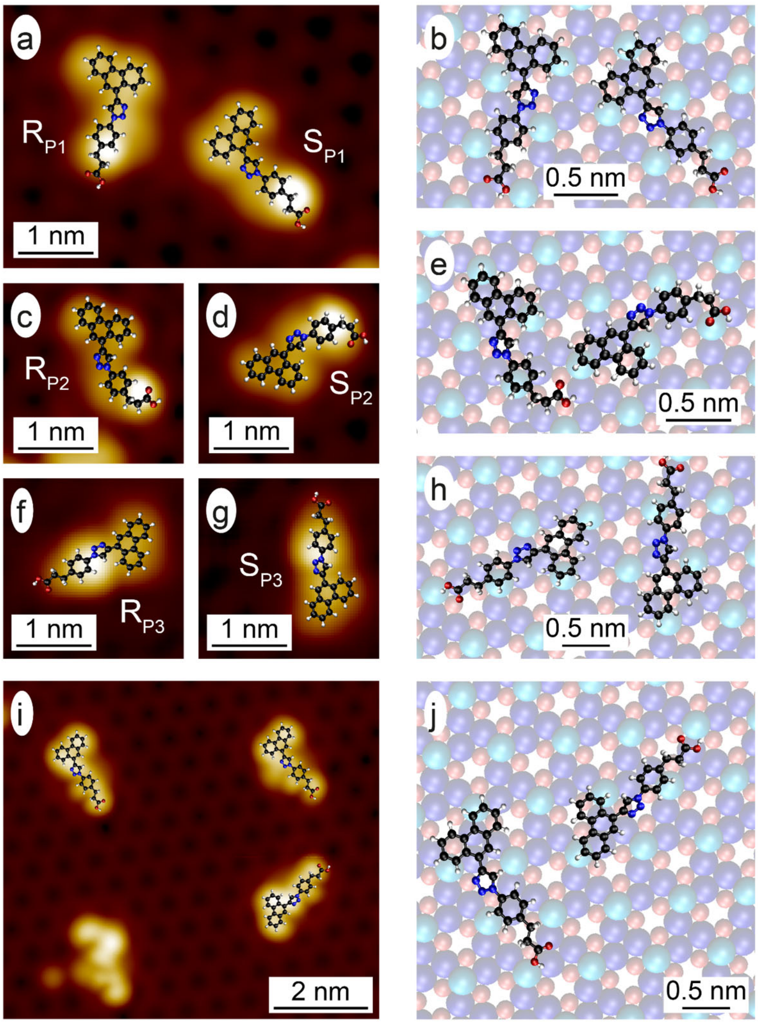

Figure S13
STM images of the 1,4-triazoles by on-surface synthesis between APA and 9-EP on the PdGa:B($\bar{1}\bar{1}\bar{1}$)Pd$_1$ surface overlaid with the molecular structures in **a**, **c-d**, **f-g** and the corresponding adsorption configurations in **b**, **e**, **h. i** STM images of the ex-situ synthesized 1,4-triazoles and **j** the corresponding adsorption geometries.
The adsorption geometries of the ex-situ synthesized 1,4-triazoles in **j** correspond to the ones shown in **b** and **e**.

Determination of reaction yield

Substrate	#APA / 100 nm^2	#9-EP / 100 nm^2	#1,4-triazole / 100 nm^2	Yield
A:Pd$_1$	6.07	8.04	2.24	37%
B:Pd$_1$	8.75	6.10	3.57	58%

Table S7
The number of APA and 9-EP molecules per 100 nm^2 after RT deposition on the A:Pd$_1$ and B:Pd$_1$ surfaces, respectively. The number of 1,4-triazoles per 100 nm^2 after subsequent annealing to 425 K. The reaction yield is determined by the formula: $Yield = 100\% * \frac{actual\ yield}{yield} = 100\% * \frac{\min(APA/100nm^2, 9-EP/100nm^2)}{1,4-triazole/100nm^2}$.

Supplementary References

1. Bebensee, F. *et al.* On-Surface Azide–Alkyne Cycloaddition on Cu(111): Does It “Click” in Ultrahigh Vacuum? *J. Am. Chem. Soc.* **135**, 2136–2139 (2013).
2. Liu, L. *et al.* α-Diazo Ketones in On-Surface Chemistry. *J Am Chem Soc* **140**, 6000–6005 (2018).
3. Gao, H.-Y. *et al.* Decarboxylative Polymerization of 2,6-Naphthalenedicarboxylic Acid at Surfaces. *J Am Chem Soc* **136**, 9658–9663 (2014).
4. Wasio, N. A. *et al.* Self-assembly of hydrogen-bonded two-dimensional quasicrystals. *Nature* **507**, 86–89 (2014).
5. Franke, M. *et al.* Temperature-dependent reaction of phthalic acid on Ag(100). *J. Phys. Chem. C* **119**, 23580–23585 (2015).
6. Morchutt, C. *et al.* Interplay of Chemical and Electronic Structure on the Single-Molecule Level in 2D Polymerization. *ACS Nano* **10**, 11511–11518 (2016).
7. Lipton-Duffin, J., Abyazisani, M. & MacLeod, J. Periodic and nonperiodic chiral self-assembled networks from 1,3,5-benzenetricarboxylic acid on Ag(111). *Chem. Commun.* **54**, 8316–8319 (2018).
8. Abyazisani, M., Bradford, J., Motta, N., Lipton-Duffin, J. & MacLeod, J. Adsorption and Reactivity of Pyridine Dicarboxylic Acid on Cu(111). *J Phys Chem C* **122**, 17836–17845 (2018).
9. Abyazisani, M., Bradford, J., Motta, N., Lipton-Duffin, J. & MacLeod, J. Adsorption, Deprotonation , and Decarboxylation of Isophthalic Acid on Cu(111). *Langmuir* **35**, 7112–7120 (2019).
